# How Does Household Residential Instability Influence Child Health Outcomes? A Quantile Analysis

**DOI:** 10.3390/ijerph16214189

**Published:** 2019-10-29

**Authors:** Emma Baker, Ngoc Thien Anh Pham, Lyrian Daniel, Rebecca Bentley

**Affiliations:** 1 School of Architecture and Built Environment, The University of Adelaide, Adelaide 5005, Australia; ngoc.t.pham@adelaide.edu.au (N.T.A.P.); lyrian.daniel@adelaide.edu.au (L.D.); 2 Centre for Health Equity, Melbourne School of Population and Global Health, The University of Melbourne, Melbourne 3053, Australia; brj@unimelb.edu.au

**Keywords:** housing, children, health, PedsQL, residential instability

## Abstract

At the core of housing and welfare research is a premise that stable residential environments are important to children’s health and development. The relationship between housing stability and health outcomes for children is, however, complex; stable housing situations are sometimes associated with poorer health outcomes, and some children may be more or less resilient to residential instability. The Longitudinal Study of Australian Children (LSAC) dataset enables us to longitudinally follow the housing and health of more than 10,000 children and their families. We employ a quantile analysis technique, a currently underutilized tool for testing associations across the distribution of an outcome, to test whether exposure to housing instability has a differential impact on children’s health dependent on their initial health status. Our findings suggest that the health outcomes of residential instability are highly dependent on children’s initial health status.

## 1. Introduction

The relationship between housing and health is now well established, with a solid body of increasingly sophisticated research exploring the diversity of ways that housing (for example its quality, location, affordability, and appropriateness) affects health (across for example mental, physical, or service usage). This work is important in the formulation of policies and interventions that harness housing to improve, protect, and maximize the health of individuals and their families. The role of housing in driving children’s health outcomes is particularly important, not only because children generally have a higher exposure to their housing (i.e., often spend more time each day at home than adults), but because this exposure is occurring at a time when lifelong developmental and health effects are formed.

Social determinants like housing [1], alongside other physical environmental features [2], are increasingly revealed as essential to the later-life health and development of children (as described in Reference [3]). Reinforcing this, consideration of the living conditions of children is highlighted as a central feature of the World Health Organization’s position on the social determinants of health. Wilkinson and Marmot’s (2003) influential work stated that “the foundations of adult health are laid in early childhood and before birth” [4]. The World Health Organization’s position on childhood as a critical period within the lifecycle sits on a strong base of neurobiological evidence, which suggests that environmental exposures during childhood are carried into adulthood [5].

The housing characteristics of children have been shown to influence their immediate (childhood), and later (adult) health via a number of direct and indirect pathways. One pathway of particular interest, links the stability of children’s housing to their current and eventual adult health and wellbeing. The residential instability of frequent house moves may, for example, affect children by cutting social connections to friends and support networks, fragment their education by moving schools, or be a direct source of emotional stress. All of these changes are likely to have educational, developmental, or health impacts, either in childhood or in subsequent adult life. Within the evidence base, a number of studies have examined these effects. Cutts et al. [6], using a cross-sectional dataset of children and their low-income families in the US, reported that multiple residential moves were associated with fair or poor health, developmental risk and lower weight-for-age scores in young children. Fowler et al. [7] found that housing mobility affected the level of behavioral problems among children. Other work by Fowler et al. [8] found that housing mobility in the prior 12 months predicted lower cognitive ability among children. At the highly precarious end of housing instability, Desmond and Kimbro [9] found that mothers who were evicted reported worse health for themselves and their children using longitudinal data from cities across the US. The systematic review by Jelleyman and Spencer [10] aimed to test the hypothesis that residential instability experienced during childhood, results in adverse health outcomes, over and above socioeconomic and other contributing factors. Overall, they found a number of health-related outcomes (such as behavioral and emotional problems, depression and illicit drug use) were associated with children’s cumulative residential mobility and instability over their lifetime. Jelleyman and Spencer concluded (following Reference [11]) that “increased residential mobility is a predictor for social, emotional and behavioral problems after controlling for family characteristics” (p. 590). 

It is important, in surveying this evidence base, to acknowledge that not all studies find evidence of a relationship between residential mobility and child outcomes (for example, Reference [12]). This is unsurprising, as some moves, even frequent moves, may be undertaken or result in, improved residential circumstances for children. Sitting parallel to the studies above, there exists a body of work that frames residential moves as potentially positive interventions; for example, moving families from disadvantaged areas to more advantaged areas—sometime referred to as ‘poverty de-concentration’—to improve educational opportunities, social relations, and health and wellbeing outcomes ([13,14,15]. One of the most well-known programs of this kind is the Moving to Opportunity for Fair Housing (MTO) experiment undertaken in the USA. In the 1990s, low-income families across five USA cities were offered vouchers to move to lower-poverty neighborhoods (*n* = 3165, control = 1439). Early evaluation of the MTO experiment found, for example, increased safety [16], improved general and mental health among household heads and fewer behavioral and psychological problems among male children [16,17], as well as lower rates of injuries, asthma attacks and experience of crime among children who moved [16]. More recent work examining longer-term outcomes [18] found that moving to more advantaged neighborhoods during childhood (i.e., 12 years old or younger) increased eventual college attendance and earnings, while also reducing single parenthood rates. Interestingly, this study also suggested that the age that children moved was important, and that moves made in adolescence were associated with modest negative child outcomes. Overall, this literature indicates that some moves can be positive, desired and can lead to better short- and long-term outcomes for both children and their parents.

The broader residential mobility literature (often concerned with why people move, where they move to, and the choices and constraints driving mobility) provides a number of additional insights. Within this field, moving houses is generally considered as a normal and regular part of a lifecycle, whereby people change residence to accommodate their changing requirements as they grow up, leave home, have children, retire and then downsize [19]. To assess if moves are successful or unsuccessful, we often look to the reasons that people provide for moving. A well-established literature classifies residential mobility in terms of the degree to which it is driven by combinations of ‘choice’ or ‘constraint’ (for example, Reference [20]). Some residential moves, for example, are driven by the desire of the household to maximize the positive aspects of their residential situation, some moves are more constrained, and may be either forced, or “induced” [21] by changes such as divorce. In the case of forced or induced moves, ‘constraint’ factors are likely to be stronger than ‘choice’ factors [22,23]. The interplay of choice vs. constraint factors may determine how successful a move is. For example, a study examining the housing trajectories of households moving from affordable or unaffordable housing found that households moving from unaffordable housing tended to relocate to less advantaged areas, while people moving from affordable housing tended to relocate to more advantaged areas [24]. So, in this case, unaffordable housing constrains, and conversely affordable housing provides choice. This evidence base as a whole reflects the variety of possible mobility pathways that children may have: some moves will be made due to choice, some due to constraint, and the great majority will have been made due to a combination of choice and constraint.

Residential moves have adverse outcomes for some children (across, for example, attention problems [25], adolescent depression [10]), but not for others (for example Reference [26]). This suggests that the pathways of effect are not simple generalized cause and outcome. The literature investigating the differing influence of housing instability on children that is depending on initial health status has yet to be adequately studied. We hypothesize that while residential instability is a potentially powerful exposure capable of driving worse health outcomes for children, it is differentially effective; that is, some children are especially vulnerable to health effects of residential instability, and some are relatively protected. Our recent analysis of the mental health effects of unaffordable housing [27] found that the effect of entering unaffordable housing was highly dependent on initial mental health. In this study, people with high initial mental health had no detectable mental health effect of entering unaffordable housing, but people with lower levels of initial mental health had significant and substantially larger effect sizes. This is important because it suggests that hidden with the traditional reporting of average effects, there are people within populations that are particularly vulnerable. The study concluded that existing mental health was protective when people were exposed to unaffordable housing circumstances. Applying this conceptual thinking to the question of the effect of residential instability on children’s health, we propose that children’s existing health may also be protective when children are exposed to residential instability.

To test whether the impact of residential instability on children’s health varies by their underlying health characteristics, we employ a quantile treatment regression approach [28]. We frame the investigation around two research questions:

1. Is there a relationship between residential instability and children’s health?
2. If so, does this effect vary by children’s initial health?

## 2. Materials and Methods

This study utilizes the Longitudinal Survey of Australian Children (LSAC) dataset. The LSAC is a linked panel dataset that has followed approximately 10,000 Australian children and their families every two years since 2003. The survey data is then linked to broader administrative data describing the children’s education, health and wellbeing. At wave 1, the dataset was designed as a nationally representative sample of Australian children. It contains a wide range of questions about development and wellbeing over the life course for two cohorts. The B cohort (“Baby”) of 5107 children was born between March 2003 and February 2004 (infants in the first survey wave), and the K cohort (“Kinder”) of 4983 children was born between March 1999 and February 2000 (children aged 4–5 years in the first survey wave). Our study uses seven biennial waves of LSAC (i.e., waves 1–7). At the most recent wave (7), children in the study were aged between 12 and 17 years. After excluding all missing values our sample contains 25,243 observations (about 43% of the LSAC data).

*Outcome variable:* The analysis aimed to test the effect of residential instability on child health across two standardized measures—physical and psycho-social health. These measures were available as summary scores from the PedsQL Pediatric Quality of Life Inventory 4.0 (as described in Reference [29]). The PedsQL is a standardized suite of child-focused health measures summarizing multiple domains of physical, social, schooling, emotional, and overall health. The data was derived from parent assessments across a number of component indicators. For each, a low score on either measure indicates a low level of health, and there is a potential range of scores from 0–100.

*Exposure variable:* Residential instability is most commonly defined as occurring when a household relocates multiple times within a short period (for example, References [30,31,32]). Previous analyses of residential instability have for example defined children’s residential instability as occurring when households made three or more moves in the first 7 years of a child’s life [30], 2 or more moves before the age of 9 [32], 2 or more times within a two-year period [33]. Other studies have not specified instability, rather focusing on the relative number of moves undertaken (for example, References [34,35]) Residential instability in this analysis is defined as occurring when a child’s family undertook two or moves during a two year period. 

To capture this instability in the biannual LSAC survey we defined instability as occurring when a child’s household moved 2 or more times between survey waves. The reference category was households who had not move or moved only 1 time between biannual waves.

*Confounders:* Potential confounders were identified from existing literature and consideration of the likely relationship between residential instability and child health. All models were controlled for child age (in months), child illness and disability, household structure, parental mental health, parent education and employment status, household income and hardship, geographical remoteness and location.

*Methods*: The classical panel fixed-effects model that relates health summary scores to residential instability is:

(1) $$health_{it}=\beta *instablity_{it}+z_{it}^{\prime }\gamma +\alpha_{i}+\mu_{t}+e_{it}$$

where $health_{it}$ represents the two child health scores i at wave t (i.e., physical and psycho-social); $instability_{it}$ is an indicator variable of whether a child i’s household at wave t moves house at least two times between waves (i.e., within the last 2 years); $z_{it}$ is a vector of the confounding variables described above; $\alpha_{i}$ and $\mu_{t}$ are unobservable child and wave fixed-effects respectively; and $e_{it}$ is the unobservable error term.

Panel regression models with child and wave fixed-effects estimators, and robust clustered standard errors were run in Stata MP 16.0 (StataCorp LLC, College Station, Texas, USA). The inclusion of child fixed-effects estimators enabled an exclusion of time-invariant characteristics, which were correlated with household residential instability and child health. As an example, in LSAC data, the primary caregiver (referred to in the dataset as Parent 1) answered the component questions from which the summary scores were derived. These responses can be correlated with the personality type of parent 1 and the child. Furthermore, personality type can be one factor contributing to a decision to move house often. For example, parents find that their child did not adapt well at school as well as neighborhood due to some unobservable characteristics of the child that are time-invariant. The inclusion of fixed-effects will enable us to detect changes in child development self-reporting and account for this form of time-invariant confounding.

We included wave fixed-effects in our longitudinal regressions. As comparisons were made for the same child at different time points, only factors that varied over time were considered for confounder adjustment. As such, the coefficients obtained from the models reflected only the differences within children who were exposed to residential instability across the survey.

The quantile fixed-effects approach of Equation (1) is:

(2) $$health_{it}=\beta \left(U_{it}\right)*instablity_{it}+z_{it}^{\prime }\gamma \left(U_{it}\right)+\mu_{t}\left(U_{it}\right)$$

and

(3) $$U_{it}=f\left(\alpha_{i},v_{it}\right)$$

for all quantiles $\tau \in \left(0,1\right)$ of the health summary scores. The effect of residential instability at the τ−quantile of the summary health scores of child i in wave t is capture by the coefficient $\beta \left(U_{it}\right)$; $z_{it}$ is a vector of control variables as discussed above; $\gamma \left(U_{it}\right)$ measures the effect of a change in the control variables at the τ−quantile of the health summary scores; and $\mu_{t}\left(U_{it}\right)$ is the year effect at the τ−quantile. The term $U_{it}$ in Equation (3) is a function of child time-invariant fixed effect $\alpha_{i}$ and time-variant idiosyncratic error $v_{it}$. This is worth noting that the form of $f\left(.\right)$ is unspecified. Clearly, the model depicted by Equations (2) and (3) does not impose a specific structure on child fixed effect. This increases the generalisation of the estimation, as in Reference [28]. We use the Markov Chain Monte Carlo algorithm (MCMC) and adjust for clustering at the individual level, as suggested in Reference [28]. 

## 3. Results

**Figure A1:**
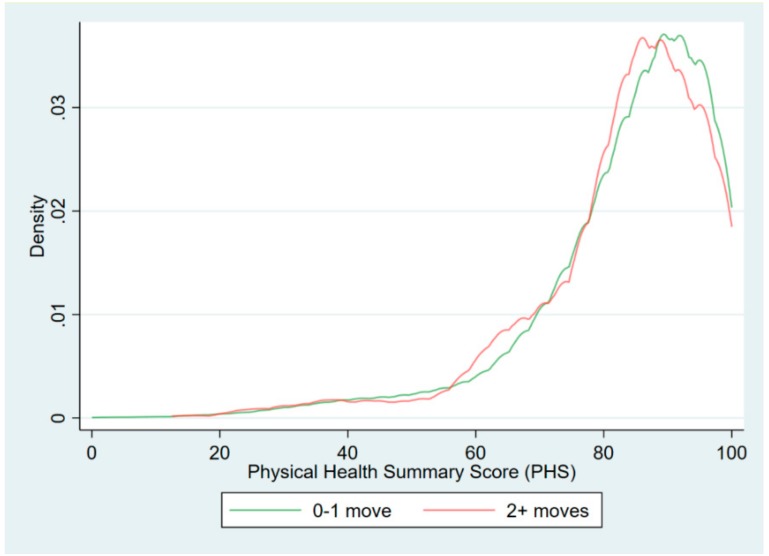
Distribution of Physical Health Summary Score.

**Figure A2:**
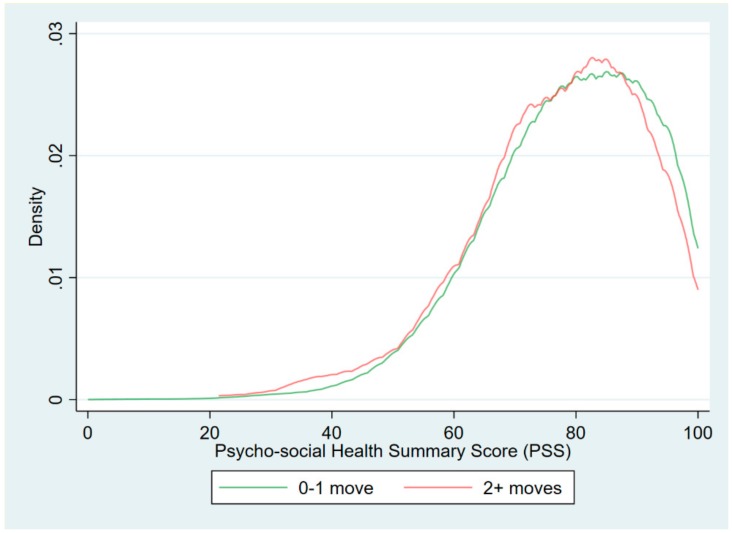
Distribution of Psycho-social Health Summary Score.

*Descriptive results:*Table 1 reports descriptive statistics (i.e., mean and difference) of the main variables of interest across residential instability status (0, 1 and 2+ moves). In the pooled cross wave dataset, residential instability was identified in around 3% of child observations, a further 17% of observations involved a single residential move, and the remaining 80% involved no residential move between waves. Overall, mean health summary scores were related to mobility level, with residential instability associated with lower health scores. On average, higher residential instability was associated with younger aged children. The majority of parents in the dataset were employed, although residential instability appears to be associated with the primary caregiver not participating in the labor force. Average household weekly income was highest for households where children made no residential moves, and lowest for households classified as residentially unstable. Regardless of residential mobility level, roughly 10% of households were in the first (lowest) income quintile, and almost a quarter were in the highest income quintile. We note that the proportion of ‘stable’ households who owned or were purchasing their homes (87%, not reported) was substantially higher than would be predicted by Australian averages (66% [36]). Tenure varied significantly by relative stability, with no moves dominantly associated with home ownership, and instability associated with rental. This is somewhat unsurprising in the Australian context, where rental contracts are typically of a 12-month duration. Similar to the distribution of the Australian population, sample households were dominantly located in cities and major regional centers. Most households reported that their dwellings are in fair or good condition (around 98%). The dwelling condition is slightly better for the highest residential instability (98.7%). The unconditional distribution of physical and psycho-social health summary scores is shown in Figure A1 and Figure A2 in the Appendix A.

*Analytical results:*Table 2 summarizes the results for the fixed-effects regression models. Notably, both effect sizes are almost uniformly small (but negative). Confidence intervals, however, suggest no statistical significance for the physical, and moderate significance for the psycho-social summary measure (at a 10% significance level).

Table 3 reports quantile treatment effects, that is, the differential effects of residential instability on children’s health outcomes, by quantile of initial health score—(lowest) 5%; 25%; 50%; 75%; 95% (highest) quintiles. Differentiated patterns of effect are clearly evidenced across the two health outcomes. The physical health results show a statistically significant physical health effect of residential instability, but this was only experienced by children with the lowest initial physical health, and for these children the effect was positive and substantial (2.952). The psycho-social health results were almost uniformly all significant and negative. Children with the lowest initial psycho-social health had the largest negative effect of residential instability.

## 4. Discussion

This paper has sought to contribute to our understanding of the mechanisms by which residential stability (or instability) affects the health and wellbeing of children. Our review of the literature highlighted the complexity of interpreting the body of existing evidence around the effects of residential instability on children’s health outcomes and suggested that there is a need to look within average effects to understand individual vulnerability. We hypothesized that a component of the variations in outcomes across studies may be due to differences in the vulnerability (or relative protection) of children, to these health outcomes. We therefore proposed that existing health characteristics may be differentially protective when children are exposed to residential instability. To test this, we applied a quantile regression approach to explore the effect of residential instability on children’s physical and psycho-social health.

At the most simple level, our research suggests that there is a relationship between residential instability and children’s psycho-social health, but not physical health. The negative effect on psycho-social health, particularly for those children who were at the poorest health initially, gives interesting evidence of residential instability potentially reinforcing health inequalities in children [37]. When initial health is taken into account in the quantile analysis, the results for physical health are particularly interesting. The only significant and appreciable effect on physical health is seen among children who have the lowest initial physical health. Our interpretation of this finding is that, for children with the lowest initial physical health, the change in housing circumstances from residential mobility motivations and the fact that some moves result in better residential conditions. We suggest that this may reflect the diversity of residential mobility and the existence of ‘choice’ motivated moves. Even though conceptually residential instability is most often regarded as a ‘risk’ [7,11] to children’s health and wellbeing, some forms of instability are clearly positive—the result of improvements in either the quality of housing or location, or improvements to children’s social setting (for example moves for the household to enter homeownership, or moves made to access more advantaged areas or better schools). A different picture emerges when we examine the quantile results for psycho-social health. Overall, psycho-social effects were negative, particularly for children in the lowest 50% of the distribution. We interpret this as children with low initial psycho-social health being more vulnerable to the insecurity and uncertainty of residential instability.

## 5. Conclusions

The application of a quantile regression approach has provided us with additional insight into the effects of residential instability on children. In this analysis, we have shown that apparent outcomes of residential instability are highly differentiated by children’s initial health and wellbeing, but in doing so have reinforced the need to ‘look within the averages’ in our data, to identify who is most at risk of poorer outcomes. In addition, following Reference [12], our analysis highlights the probability that a great proportion of moves made by families may be positive (for example moving house to facilitate entry into homeownership, or movement to enroll children in a closer school). This means that any result for the effect of residential instability on child health seen in our analysis (and previous similar analyses) is potentially ‘softened’ by an averaging with positive outcomes of those moves. This means that effect sizes for children across the literature are likely to be much larger.

Finally, our analysis suggests that much remains to be done to better understand the pathways of effect. Key among the ideas to build and refine this analysis is the incorporation of a more nuanced understanding of mobility within the models. Previous work (and this current analysis) has been framed within an understanding of residential instability as a more or less one-dimensional exposure of stable/unstable. The challenge for subsequent research is to incorporate an understanding of residential instability not just as a simple exposure, but as one that captures why families move.

## Figures and Tables

**Table 1:** Summary statistics of selected groups of 0, 1 and 2+ moves.

Variables	Number of House Moves					
	0	1	2+ (Instability)	Mean Diff. 0 vs. 1 Move	Mean Diff. 1 vs. 2+ Move	Mean Diff. 0 vs. 2+ Move
Children (observations)	20,281	4199	763			
Health Summary Scores (mean)						
Physical	83.6	83.3	82.9			
Psycho-social	79.1	78.6	77.2	**	**	***
Cohort (%)						
Birth	53.4	60.3	63.2	***		***
Kinder	46.6	39.7	36.8	***		***
Average age of children (years)	8.5	7.5	6.7	***	***	***
Primary care giver—Labour force status						
Employed	77.7	70.1	65.5	***	**	***
Unemployed	1.6	2.4	2.8	***		
Not in labor force	20.7	27.5	31.7	***	**	***
Parent 2 Labour force status						
Employed	95.6	94.4	93.4	***		**
Unemployed	1.3	1.8	2.2	**		
Not in labor force	3.1	3.8	4.3	**		
Household weekly income ($ average)	2397.2	2326.0	2207.0	***	**	***
Income quintile (%)						
1	10.4	10.7	10.1			
2	19.2	19.4	19.7			
3	22.5	22.0	21.8			
4	24.0	22.2	25.0	**	*	
5	23.9	25.8	23.5	***		
Tenure (%)						
Home owner	91.7	65.0	58.7	***	***	***
Public renter	0.9	0.7	1.7		**	*
Private renter	4.1	23.8	27.9	***	**	***
Remoteness (%)						
City	87.3	86.2	85.7	*		
Regional	11.2	11.6	11.7			
Remote	1.4	2.1	2.6	***		*
Dwelling in fair/good condition (%)	98.0	98.3	98.7			

Note: * *p* < 0.1, ** *p* < 0.05, *** *p* < 0.01.

**Table 2:** Summary of fixed effect models.

Variable	Health Summary Score	
	Physical [LCI; UCI]	Psycho-Social [LCI; UCI]
Residential instability	−0.534	−0.805 *
	[−1.541; 0.472]	[−1.686; 0.077]

Note: * *p* < 0.1, ** *p* < 0.05, *** *p* < 0.01. 95% confidence intervals are reported in the brackets. Standard errors are clustered at the child level. All regressions are controlled for childrens’ characteristics (age, child disability or illness, live with 2 parents), parental characteristics (depression, education, employment, household income, hardship score), state/territory, remoteness, individual and wave fixed-effects.

**Table 3:** Summary of quantile treatment effect of residential instability models.

Quantile Treatment Effect				
0.05	0.25	0.5	0.75	0.95
Physical Health Summary Score (PHS)				
2.952 **	0.241	−0.197	−0.119	0.000 ***
[0.479; 5.425]	[−0.421; 0.903]	[−0.606; 0.212]	[−0.683; 0.444]	[0.000; 0.000]
Psycho-Social Health Score (PSS)				
−2.284 ***	−0.630 ***	−1.905 ***	0.141	−0.332 **
[−2.864; −1.703]	[−0.997; −0.263]	[−2.849; −0.962]	[−0.078; 0.360]	[−0.638; −0.026]

Note: * *p* < 0.1, ** *p* < 0.05, *** *p* < 0.01. 95% confidence intervals are reported in the brackets. Standard errors are clustered at the child level. All regressions are controlled for children characteristics (age, child disability or illness, live with 2 parents), parental characteristics (depression, education, employment, household income, hardship score), state/territory, remoteness, individual and wave fixed-effects.

## Appendix A

Figure A1 and Figure A2 show the empirical density functions of physical and psycho-social health summary scores for different population groups. The distributions of the two health outcomes are different between children that were exposed to 0–1 move and 2+ moves (i.e., residential instability group). Both figures show that the mass of the distributions for the 2+ moves group is less concentrated on the right than that of the 0–1 move group.
